# Perceptions and preferences for genetic testing for sickle cell disease or trait: a qualitative study in Cameroon, Ghana and Tanzania

**DOI:** 10.1038/s41431-024-01553-7

**Published:** 2024-02-19

**Authors:** Nchangwi Syntia Munung, Karen Kengne Kamga, Marsha J Treadwell, Jemima Dennis-Antwi, Kofi A Anie, Daima Bukini, Julie Makani, Ambroise Wonkam

**Affiliations:** 1 Division of Human Genetics, University of Cape Town, Capetown, South Africa; 2 Medical Genetic Service, Regional Hospital Limbe, Limbe, Cameroon; 3 University of California San Francisco Department of Pediatrics/Division of Hematology, Oakland, CA USA; 4 Centre for Health Development and Research, Accra, Ghana; 5 London Northwest University HealthCare (NHS) Trust, London, UK; 6 Imperial College London, London, UK; 7 Muhimbili University of Health and Allied Sciences, Dar es Salaam, Tanzania; 8 McKusick-Nathans Institute & Department of Genetic Medicine, Johns Hopkins University School of Medicine, Baltimore, MD USA

**Keywords:** Ethics, Anaemia

## Abstract

Sickle cell disease (SCD) is a single gene blood disorder characterised by frequent episodes of pain, chronic anaemic, acute chest syndrome, severe disease complications and lifelong debilitating multi-system organ damage. Genetic testing and screening programs for SCD and the sickle cell trait (SCT) are valuable for early diagnosis and management of children living with SCD, and in the identification of carriers of SCT. People with SCT are for the most part asymptomatic and mainly identified as through genetic testing or when they have a child with SCD. This qualitative study explored perceptions towards genetic testing for SCD and SCT in Cameroon, Ghana, and Tanzania. The results show a general preference for newborn screening for SCD over prenatal and premarital/preconception testing, primarily due to its simpler decision-making process and lower risk for stigmatization. Premarital testing for SCT was perceived to be of low public health value, as couples are unlikely to alter their marriage plans despite being aware of their risk of having a child with SCD. Adolescents were identified as a more suitable population for SCT testing. In the case of prenatal testing, major concerns were centred on cultural, religious, and personal values on pregnancy termination. The study revealed a gender dimension to SCD/SCT testing. Participants mentionned that women bear a heightened burden of decision making in SCD/SCT testing, face a higher risk of rejection by potential in-laws/partners if the carriers of SCT, as well as the possibility of  divorce if they have a child with SCD. The study highlights the complex cultural, ethical, religious and social dynamics surrounding genetic testing for SCD and emphasises the need for public education on SCD and the necessity of incorporating genetic and psychosocial counselling into SCD/SCT testing programs.

## Introduction

Genetic testing for sickle cell disease (SCD) and the sickle cell trait (SCT) is not a routine component of public health programs in Africa, even in countries with a high SCD burden. SCD is an inherited autosomal recessive blood disorder caused by genetic mutations in the haemoglobin gene, leading to the production of sickle or banana-shaped red blood cells (RBC) instead of the normal biconcave-shaped RBC. People living with SCD often experience severe anaemia, frequent episodes of pain, infections, stroke, acute chest syndrome, severe disease complications and lifelong debilitating multi-system organ damag [1]. On the other hand, individuals with SCT are for the most part asymptomatic, though in rare cases may have a painful crisis. Also the SCT maybe a risk factor for some common SCD complications [2, 3].

Africa bears the highest global burden of SCD with countries like Cameroon, Ghana, Nigeria, Tanzania and The Democratic Republic of Congo (DRC) reporting an SCD prevalence of between 1–3%; and a  20–30% incidence of SCT [4–6]. Approximately 405,000 babies are born with SCD each year in sub-Saharan Africa, constituting about 79% of the annual SCD births worldwide [6]. The highest mortality burden, including under 5 mortalities from SCD, is also concentrated in Africa [6]. Given the high burden of SCD in Africa and the substantial economic, social, and psychological costs, genetic testing and screening programs for SCD and SCT may be potentially effective for managing the disease [7, 8]. Three recommended genetic testing programs are new-born screening (NBS), premarital/preconception screening for SCT, and prenatal screening. NBS facilitates early diagnosis, potentially leading to timely and improved infant management. Prenatal screening allows at-risk couples to determine if their unborn child will have SCD, informing decisions on pregnancy, including medical termination or early SCD care and management. Premarital screening identifies carriers of the SCT, aiding reproductive planning and decision making.

Universal screening for SCD and SCT is limited in Africa, with Egypt being the sole African country implementing universal NBS for SCD. Some countries like Angola, Benin, Ghana, Nigeria, Zambia have piloted NBS programs for SCD [9–14]. Cameroon had previously piloted prenatal testing for SCD as part of a research initiative to introduce medical genetic services at a tertiary health facility [11, 15, 16], and some states in Nigeria are considering bills for mandatory premarital testing and registration of new births [17].

While genetic testing for SCD and SCT offers many public health benefits, the psychological impact on affected individuals and the influence of socio-cultural and religious factors on the decision to test for SCD/SCT are important considerations [18–21], as they are likely to play a pivotal role in public acceptability of SCD/SCT screening programs. In this paper, we present the outcomes of a qualitative study on perceptions and attitudes towards genetic testing/screening for SCD and SCT among individuals living with SCD and their caregivers. This included perceptions  and attitudes towards premarital/preconception, prenatal, and newborn screening. This study is part of broader research project on stakeholder’s perceptions of public health genomics and SCD interventions in Africa, with the study sites being Cameroon, Ghana, and Tanzania [22].

## Method

### Study design and participants

This was a qualitative cross-sectional study that primarily used focus group discussions (FGDs) for data collection. We occasionally resorted to individual in-depth interviews (IDIs) when faced with logistical challenges such as participant availability, willingness of family members to participate in the study and age considerations for informed consent. FGDs are a widely recognized qualitative methodological approach for capturing diverse perspectives and gathering rich qualitative data [23]. Study participants were individuals directly impacted by SCD, either as patients or family members and who had been engaged in an SCD screening or testing program. Purposive sampling [24] was used to select participants. The lead investigator in each country were healthcare workers and researchers who had direct experience in managing SCD clinics or in leading SCD research projects [25] and they played a central role in identifying potential participants.

### Study setting

This study was conducted in Cameroon, Ghana and Tanzania, geographically representing Central, West and East Africa respectively. The estimated prevalence of SCD in the three countries is approximately 2%, with 20–30% of the general population carrying the SCT [12, 26, 27]. SCD is a significant contributor to the under-five mortality rate in these countries and none have routine public health screening programs for SCD or the SCT. Costing analyses for SCD screening in Africa is sparse but estimates for point of care testing for SCD using isoelectric focussing, suggest it may range from 5 to 19 USD [28–30]. In all three countries, prenatal testing is mainly available in private health facilities and at a high cost.

### Data collection

The primary data collection method for this study was FGD. The FGDs were conducted in multiple languages to accommodate the language preferences of participants across the different sites, specifically in French in Cameroon, Twi in Ghana, Swahili in Tanzania, and English at all the sites. A combination of a FGD guide and vignettes [31] was used for the FGDs. The FGD guide provided a structured framework for the discussions, while the vignettes, depicting a couple facing decision-making about genomics and public health interventions for SCD, added a contextual and personalised dimension to the conversation. The vignettes facilitated exploration of perceptions regarding newborn screening, premarital screening, prenatal diagnosis, genetic counselling, possibility of stigmatization; and strategies for enhancing uptake of SCD public health genetic interventions [22].

Participants in Ghana and Tanzania were 18 years or older, while in Cameroon, the age criterion was 21 years or older, aligning with national definitions for adulthood [32]. All participants provided written informed consent, and ethical approval was obtained from research ethics committees at the participating institutions, namely: Muhimbili University of Health and Allied Sciences, Tanzania; University of Yaoundé, Cameroon; Kwame Nkrumah University, Ghana; and the Faculty of Health Sciences, University of Cape Town, South Africa.

### Data analysis

The FGDs and IDIs were digitally recorded and subsequently transcribed verbatim. Where data was collected in languages other than English, the transcripts were translated to English to facilitate analysis. All transcripts were imported into NVivo for deductive thematic analysis [33]. A coding scheme consisting of four thematic areas: “acceptability of screening”, “barriers for screening”, “benefits of screening,” and “decision making for testing” was applied to all the transcripts. These thematic areas were derived from previous formative research on NBS for SCD in Ghana [22]. Researchers from the respective study sites undertook the coding process using the agreed-upon coding scheme. Discrepancies in coding were resolved through consensus. The methodology and tools employed in this study have been detailed in a methods paper for the larger study [22] and an, subsequent publications on consent for genetic studies involving paediatric populations [34] and SCD related stigma [25]. In presenting the results, SCD-PT denotes an individual living with SCD, while SCD-FPT signifies family member of a person living with SCD.

## Results

The study engaged a total of 102 individuals living with SCD and their families through 19 FGDs and IDIs (Table 1). Each session lasted 90–120 min.

**Table 1:** Demographics and characteristics of individuals with scd and their family members in Cameroon and Ghana.

	Demographics	Cameroon (*n* = 25)	Ghana (*n* = 60)	Tanzania (*n* = 17)
Reported gender	Female	12	37	–
	Male	21	11	–
Educational level	Primary	08	4	–
	Secondary	10	40	–
	Tertiary	2	13	–
Data Collection method	FGD	03	06	02
	IDI	03	01	04

n: total number of participants; -: information not available. Total umbers may not add up as some participants did not provide demographic information. Demographic data were not consistently collected from Tanzania and are not presented [19].

Overall, the findings revealed hesitancy towards premarital and prenatal testing for SCT or SCD, while NBS was perceived as more socially acceptable and of high public heath relevance. Participants highlighted several factors that could influence public acceptability of SCD screening and testing programs, such as religion/ faith-based values, cultural beliefs; perceived public health value of SCD screening; cost of screening; public awareness of SCD; and the availability of SCD support programs before and after testing (Table 2).

**Table 2:** Factors that may influence acceptability of SCD/SCT testing programs.

Factors that may influence acceptability of SCD screening programs	Premarital/preconception screening	Prenatal screening	Newborn screening
Perceived social value	Overall limited social value	Overall limited social value	High social value
Challenges with decision-making	Involve couples and their parents in decision-making and counselling	Involve both parents in decision-making	Involve both parents in decision-making
Cost of testing	Prohibitive cost a limiting factor	Participants had no idea of cost	Cost is not a major limiting factor
Preference for pre and post-test counselling	Both religious leaders and parents should do counselling	Both healthcare workers and religious leaders should do counselling	Healthcare workers should do counselling
Public Awareness of screening programs	General but not extensive awareness	Limited awareness	High awareness
Faith based or religious beliefs	High	High.	Low
Willingness to comply with post-testing recommendations	Highly unwilling to cancel marriage plans	Reservations related to termination of pregnancy	High compliance to start treatment and management.
Averting reluctance for screening	Policies that mandate pre-marital screening.	Provide information and counselling to parents	Increase accessibility to NBS; information provision and counselling

### Premarital genotype screening for the sickle cell trait (SCT)

Participants had an overall positive attitude toward premarital testing for the SCT, often drawing a parallel with premarital testing for HIV.

If a person intends to get married, the test should be done so that you discover each other, just like it is done with HIV/AIDS. I will not advice even my brother to get married without testing for Sickle Cell. (SCD-FPT-Cameroon)

This positive outlook was shaped by first-hand experiences of the physical and emotional struggles associated with SCD.

I will definitely do it [SCD test] if I want to get married. I will tell her, let’s go and be tested. The first thing that will make me agree to taking this test is that I do not want my child to go through what I went through. So, we must be tested no matter how much I love her. (SCD-PT-Tanzania)

While recognising the benefits of premarital testing, various factors were listed as potential barriers to widespread adoption of premarital testing. This included the impact of testing on decisions related to marriage and childbearing.

The test is good before marriage. My husband and I had the test, and we were told not to marry but because we had made all the preparations, we went ahead and married (SCD-FPT-Ghana).

The decision to proceed with marriage and childbearing despite both couples having the SCT was influenced by deep rooted cultural and religious beliefs. For example, there was a prevalent belief amongst participants that having a child with SCD is determined by chance or a higher power (God). Additionally, the pressure to conform to social expectations of marriage and procreation may override concerns about the risk of having a child with SCD.

Let’s take two young spouses who want to get married, AS //AS [carriers]. They marry and have 7 children, none has sickle cell, why? It is the chance, right? It means that sickle cell disease is a question of chance. It is God who gives you these children. So, for me if you see a couple who love each other, and who do not really know if they will have a child with sickle cell disease; why do you forbid marriage? (SCD-PT-Cameroon)

Negative perceptions of a couple’s suitability for marriage, doubts about their ability to have a healthy family, and disapproval of marriage plans by potential in-laws were listed as potential reasons why people may not want undergo premarital/preconception testing for the SCT.

I have a fiancé; he wants to marry me. He heard about my problem [SCD] and agreed to get tested, but he fears that his relatives, mother, and father will not accept me, something which hurts me. (SCD-PT-Tanzania)

A gender dimension to undergoing premarital screening for SCT emerged from the discussions. Some participants mentionned that the burden of rejection by potential in-laws or a future spouse, following premarital testing, is particularly significant for the female partner and that women faced a higher risk of experiencing marital challenges, including the possibility of divorce, if they were to have a child with SCD.

Some men can divorce their wives when the children have SCD, so it is better to know your status and separate before marriage (SCD-PT-Cameroon)

Finally, financial constraints were identified as a barrier to accessing premarital screening, making it challenging for some couples to undergo testing and counselling on reproductive choices.

I think it could be the cost. If they find that the test costs one million and maybe, they can only afford twenty thousand. For some Tanzanians, may be twenty thousand is too big and they may not even have a shilling (SCD-PT-4 Tanzania).

To increase the acceptability of premarital screening, participants proposed the implementation of policies or laws mandating premarital screening for SCT.

I also think the government should make it a law. I have heard that in Saudi Arabia, for instance, before you get pregnant you must do the test. So, if it is a law which states that if both couples are AS they should not marry, it will reduce it. (SCD-PT--Ghana)

Another suggestion was to implement population-wide adolescent screening for the SCT, as it could empower young people to engage in early discussions with potential partners about their genotype.

It will be good if the youth of today test and have the results and not wait until it is time to marry, so when they meet someone, they are interested in, they can ask, what is your blood group, sickling and others even before they decide to be together. I gave birth to two sicklers, one is dead leaving the other. It is not easy at all. For me before we meet to marry it is good that we know so it can be prevented before marriage. (SCD-FPT-Ghana)

The above highlights the multi-faceted nature of decision-making for premarital screening for SCT and the need to consider how cultural, religious, gender, and economic factors may negatively impact on premarital screening programs for SCT.

### Prenatal genetic testing for SCD

Prenatal testing provides pregnant couples with information about their baby’s risk of having SCD. It can be performed during the first or second trimester of pregnancy through procedures such as chorionic villus sampling or amniocentesis, both of which are considered invasive. However, other non-invasive procedures for prenatal screening are being developed [35]. The FGDs revealed a general acceptance for prenatal diagnosis, especially if it poses no harm to the unborn child.

First, it depends if the test will not bring harm to the unborn child. Nowadays, most people would like to know the status of their child before they are born, and this will also help them to prepare with whatever outcome. It will also help them to know ways that they can use to take care of their child once he/she is born. (SCD-PT Tanzania)

Participants emphasized the need for comprehensive education on the procedures and importance of prenatal screening.

Sessions in the clinics that talk about sickle cell disease and adverts that explains the importance of getting tested during pregnancy. If we increase the level of awareness in the community then many people will participate (SCD PT-Tanzania)

Although participants mentioned that pregnant women may be willing to undergo prenatal testing for SCD, they also expressed uncertainty about its overall social value, suggesting that they may perceive prenatal testing as being of little public health impact. Participants’ reservations for prenatal testing were influenced by several factors. One is a lack of awareness or understanding about the purpose of prenatal testing.

What will the doctor do after getting the results? (SCD-FPT-Cameroon)

Some participants expressed concerns that prenatal testing could leave parents, especially mothers, traumatized and anxious if their unborn child is diagnosed with SCD. This is because parents may have developed emotional bonds with their unborn child and may not be willing to opt for termination of pregnancy. This situation highlights the emotional and ethical considerations that parents face when confronted with the possibility of having a child with SCD.

I will not do the test because the person is already pregnant. There will be no need for this test, no matter the outcome. The lady is already pregnant, are they going to abort or what? Can they reverse the situation if it comes out that the baby has sickle cell disease? (SCD-FPT-Ghana)

Other participants highlighted that the anxiety of having a child with SCD may be compounded by concerns about the potential impact of the results on their marital relationships. Noting that the burden of testing weighs more on the female partner. Like with premarital testing, this perspective further highlights the gender dynamics and social pressures that couples, especially the female partner, may experience when they opt for SCD/SCT screening programs.

I will not go for the test because the man will leave me when the results turn out to be positive. I will bear the sole responsibility of the pregnancy and the baby. (SCD-FPT-Ghana)

The reservations towards prenatal screening were also rooted in personal values, cultural beliefs, and religious norms on the termination of pregnancy. Some participants expressed opposition to medical termination of pregnancy arguing that advancements in medicine have made SCD a manageable condition and that the life expectancy among individuals with SCD as increased over the years.

There should be no ending of pregnancy because the bible is against it. I only need the doctor to explain if there is any medicine to give after birth. I have seen somebody with SCD who lived long. (SCD-FPT-Cameroon)

For some participants, if they were to undergo prenatal screening for SCD the intention will not be to opt for termination of pregnancy, but rather to anticipate and prepare for the specific needs and challenges associated with caring for a child with SCD.

The pregnancy has been formed. You cannot advise for abortion, it is not possible, you have to accept it so that when the child comes, you will know how to take care of him/her. I have no ability to change the status. I am ready to receive my child with the disease and take care of him. (SCD-FPT- Tanzania)

Some participants expressed uncertainty about the goal and social value of prenatal testing emphasizing that individuals with SCD can live their full potential and make valuable contributions to their families and communities. There was also the perspective that prenatal screening could perpetuate stigma and discrimination against individuals with SCD, as it may convey a message that their lives are not worth living.

You can’t deny the child a right to live because he/she has a certain problem. If my parents had done that, then perhaps I wouldn’t have been here. I believe over the years I have done something to help them. So, I think they should keep the pregnancy as they will never know who the child will become and how he/she will help them. (SCD-PT-Tanzania)

The excessive cost of prenatal testing was seen as a deterrent for adoption especially as there are little options on what parents can do if the test shows that the child will have SCD.

I hear it is expensive and it is not necessary to do the test. You are already pregnant, so it is up to God (SCD-FPT-Ghana)

The quotes above show a high inclination towards religion as a coping strategy and reflects the role of spiritual and emotional support as part of genetic testing programs.

### Newborn screening (NBS) for SCD

A number of pilot programs have demonstrated the public health benefits of NBS for SCD [36, 37]. In our study, participants expressed a more positive outlook towards NBS compared to premarital and prenatal screening or testing for SCD.

It is better to test a child when she/he is young. A good example is my parents. They knew I had sickle cell when I was a child of four months, and they started giving me good care and attending clinic well. If you don’t know that your child has sickle cell, and you refuse to take the test, you will not know and as a result the child die while still young but if you know you could have prevented the child from dying because you already know your child has sickle cell (SCD-PT-Tanzania)

Another benefit of NBS, as mentioned by participants, was that it could help prepare parents for their child’s first pain crisis and also dispel myths about the health and wellbeing of the child.

It is best if they know during the early stage as it gives them ample time to prepare for the outcome and to know what to do with the child’s condition. And with this understanding it will help to avoid the misconception about the disease from the community as the parents will know what is happening with their child. (SCD-PT-Tanzania)

Besides the benefits to the child in terms of early initiation to care, another advantage of NBS was the psychological benefit to mothers.

I always use my example. My first child was detected at six months; we found that she had sickle cell. When I delivered the second baby, I did the test at birth, so I could have some peace. But unfortunately, she was also SS. It helps because when you are aware, you know how to deal with it and the care is better than when you don’t know. (SCD-FPT-Cameroon)

Possible barriers to NBS included religious beliefs and limited awareness of SCD. To address these, it was recommended health promotion programs on SCD.

If some people do not agree, then those people might be Jehovah witnesses but apart from that everybody agree for blood to be drawn from the baby’s heel when they are born (SCD-PT-Ghana)

Those who will not agree may be due to their education level. Illiteracy might be a hindrance. They would not know the importance of the test but if they knew they wouldn’t even waste time, they would just agree for it to be done (SCD-PT-Ghana)

To increase acceptability of NBS, some paricipants recommended that genetic and/or psychosocial counselling should be integrated in all testing programs for SCD and SCT (Table 3). Such counselling, they suggested, should include comprehensive information about the benefits of NBS and  SCD support services.

They [parents] will accept to know the status of their child. If you give them awareness and make them understand about the treatment and all things concerning the disease, then there will be no problem (SCD-PT-Tanzania)

**Table 3:** Barriers and enablers for of genetic testing for SCD and SCT.

Barriers /Enablers	Premarital testing	Prenatal testing	NBS
Barriers to acceptability and adoption	Fear of rejection by potential in-laws	Religious and cultural norms on moral status of the embryo termination of pregnancy	Lack of SCD care and counselling programs at SCD clinics
	Reluctance to disappoint a potential spouse	Invasive procedure	Parents uncomfortable with drawing blood samples from new-born
	Religious and cultural beliefs the gift of a child	Restrictive national laws and regulation on termination of pregnancy	Religious beliefs in some instances
	Possibility of stigmatisation	Excessive cost of testing	
Improving Acceptability	Mandatory laws and policies testing for SCT.	Education on SCD and prenatal testing at antenatal clinics	Provide counselling especially for mothers
	Involve parents of couples and religious leaders in counselling process	Involve community and religious leaders in counselling process	Information provision on available SCD care and support fasciitis
	Population screening for SCT in adolescents	Use of non-invasive procedures	Reduce cost of testing

## Discussion

Our study showed that NBS was viewed more favourably in terms of social and public value. This is because NBS is non-invasive, does not involve complex decision-making processes based on one’s cultural and religious beliefs and there is less potential for stigmatisation. The study also highlights unique social, ethical, and cultural factors that may influence acceptability of SCD genetic testing programs in Africa (Table 3), including the need to pay attention to gender dynamics in SCD screening and testing programs as the outcome of results tend to disproportionately affect women compared to men. Furthermore, involving healthcare workers, religious leaders, community leaders when counselling couples who are at risk of having a child with SCD is key to improving adoption and acceptability.

Overall, participants were equivocal about the benefits of premarital screening and suggested that it may be more beneficial if designed as a public health screening program for adolescents, allowing them to know their haemoglobin genotype at an age when marriage or childbirth is not a primary concern. A similar approach has been used in some countries in the Middle East [38]. Fear of rejection by family members, especially potential in-laws, emerged as a significant barrier to the acceptance of premarital testing for SCD in all three countries. In Ghana, there was near unanimous support for advising at-risk couples not to proceed with their marriage plans if they were identified as carriers of the SCT, and participants considered premarital testing as a proactive measure to reduce the incidence and burden of SCD. In contrast, participants from Tanzania and Cameroon leaned more towards counselling for SCD management and care, rather than advising against marriage as it could lead to frustrations, for couples who are already committed to each other. Evaluation and perceptions of premarital screening programs in the Middle East have also shown that premarital screening for monogenic conditions was unsuccessful in discouraging at-risk marriages [39], although it may contribute to reducing the prevalence of affected births in countries that provide prenatal diagnosis and therapeutic abortion [40, 41].

There was less support for prenatal testing in all three countries. This lack of support was primarily based on religious norms surrounding the termination of pregnancy. Many participants emphasized the sanctity of life and believed that with proper medical care for SCD, positive health outcomes and improved quality of life could be achieved. Sociological surveys conducted in Cameroon and Nigeria corroborate these findings [18, 42]. Prenatal testing for SCD offers parents with either the option to terminate the pregnancy or to keep pregnancy till term and introduce SCD care after birth. However, in many African countries, laws or regulation on termination of pregnancy are highly restrictive, often allowing termination of pregnancy only when it is a danger to the woman’s health, and in rare cases, for socio-economic reasons [43]. This restrictive legislation adds complexity to the acceptability of prenatal testing. Public health programs for prenatal screening would have to consider the intricate psychosocial, legal, and ethical nuances that may arise in implementing prenatal screening, including perceptions in African settings on the moral status of the embryo and genetic selection. Investing in public education on SCD, genetic counselling training for healthcare providers may be useful for improving acceptability of genetic testing programs for SCD.

## Conclusion

The results highlight the importance of addressing social, ethical and cultural factors in implementing genetic testing and screening programs for SCD and SCT; and for education, genetic counselling and SCD awareness campaigns in fostering public understanding and acceptability of genetic testing and screening for SCD. Sensitivity to the cultural context, coupled with the provision of psychosocial support, counselling and SCD education will be crucial in mitigating public concerns and hesitancy around SCD/SCT testing. In addition, efforts should be directed towards minimising the potential of stigma and discrimination following SCD testing and screeing programs, and to consider gender disparities in the burden of decision making. Consequently, we recommend the integration of genetic counselling into SCD testing programs in the three countries. We acknowledge that there is limited capacity for genetic counselling in these countries. However, ongoing initiatives in Ghana and South Africa to train more genetic counsellors present a positive trajectory for SCD programs and genetic medicine in Africa. In the interim, SCD programs in Africa could bridge the gap in counselling services, by intergrating health and social counsellors into SCD testing programs and training them in a basic concepts in genetics and SCD.

### Study limitation

This study included SCD patients and their relatives only. It is likely that the results could be quite different if the study involved the general population and not just SCD patients and their family members. A bigger study would provide more insights into perceptions about genetic testing for SCD and the SCT.

## Data Availability

The dataset for this article is available upon reasonable request from the corresponding author.
